# How the Electrical Conductivity of Water Fluids Affects Micro-EDM in the Short-Pulse Regime

**DOI:** 10.3390/mi15020266

**Published:** 2024-02-13

**Authors:** Valeria Marrocco, Francesco Modica, Vincenzo Bellantone, Marcello Valori, Irene Fassi

**Affiliations:** 1 STIIMA-CNR Institute of Intelligent Industrial Technologies and Systems for Advanced Manufacturing, National Research Council of Italy, Via P. Lembo 38/F, 70124 Bari, Italy; francesco.modica@stiima.cnr.it (F.M.); vincenzo.bellantone@stiima.cnr.it (V.B.); 2 Technology Transfer Directorate, Italian National Agency for New Technologies, Energy and Sustainable Economic Development, Via Martiri di Monte Sole, 4, 40129 Bologna, Italy; marcello.valori@enea.it; 3 STIIMA-CNR Institute of Intelligent Industrial Technologies and Systems for Advanced Manufacturing, National Research Council of Italy, Via A. Corti 12, 20133 Milan, Italy; irene.fassi@stiima.cnr.it

**Keywords:** micro-electro discharge machining (EDM), water fluids, plasma channel, electrical conductivity, material removal, crater size

## Abstract

This work investigates micro-electro discharge machining (EDM) performance involving deionized and tap water. The chosen machining regime was semi-finishing, where open voltage (from 100 to 130 V) and current values (5–10 A) were applied using a 0.5 µs pulse-on time and a frequency of 150 kHz, i.e., a duty cycle of 25%. First, numerical analyses were performed via COMSOL Multiphysics and used to estimate the plasma channel distribution and melted material, varying the current, sparking gap, electrical conductivity, and permittivity of the two fluids. Then, experimentally, the micro-EDM of holes and channels in hardened thin steel plates were replicated three times for each considered fluid. The material removal rate (MRR), tool wear ratio (TWR), radius overcut, and surface roughness were plotted as a function of open voltage and electrical conductivity. The study proves that as voltage and current increase, the MRR and TWR decrease with electrical conductivity. Nonetheless, for higher electrical conductivity (tap water), the process did not proceed for lower open voltages and currents, and the radius overcut was reduced, contrary to what is commonly acknowledged. Finally, the crater morphology and size were evaluated using a confocal microscope and compared to simulated outcomes.

## 1. Introduction

Electro-discharge machining (EDM) is an electro-thermal process where material removal is accomplished through the erosion achieved by sparks occurring between two electrodes, i.e., the tool and the workpiece. In EDM, the discharge establishes a plasma that causes the temperature to rise, leading to both electrode materials’ melting and evaporation. EDM and its micro counterpart are contactless processes and, as such, they are capable of machining various electro-conductive materials independently of their hardness and strength [1,2,3,4], including conductive composite materials, such as conductive ceramics, characterized by brittleness [5,6]. However, some limitations for micro-EDM arise in the case of very brittle materials where the recast layer can introduce stress that may cause workpiece breakage.

During erosion, dielectric fluid is generally used for flushing to improve plasma channel confinement, increase the energy density locally within the sparking gap, promote heat transfer, and remove debris. For dielectric fluid, hydrocarbons, mineral oil, kerosene, and water-based liquids were widely investigated for classic EDM. Additionally, the addition of a variable concentration of non-conductive and electroconductive powders to such dielectric fluid was investigated to improve EDM performance [7,8]. 

In this regard, Chakraborty et al. [9] reviewed the role of various dielectric fluids, such as hydrocarbons, mineral oil, water-based liquids, and powder-mixed dielectrics used in EDM. In particular, it was generally observed that water-based media allow for a higher thermal stability and material removal rate (MRR), especially under critical conditions. Drinking water as a dielectric was the object of Gugulothu et al.’s work on die-sinking EDM of Ti-6Al-4V alloy [10]. An ANOVA analysis conducted by varying the current (10, 15, and 20 A), pulse-on time (25, 45, and 65 µs), pulse-off time (24, 36, and 48 µs), and graphite powder concentration allowed the authors to assess the effect of process parameters and various fluids on the MRR, surface roughness, and recast layer thickness. Early work in 1984 [11] showed that mixing 75% distilled water and 25% tap water when low-carbon steel workpieces are machined at low-current densities and low-voltage settings can result in a better MRR and almost zero tool wear. 

Generally, the inspection of the literature on EDM with water-based fluids leads to the conclusion that significant differences in behavior and performance must be accounted for when using electro-conductive fluids, such as water, compared to carbon-based or organic dielectrics. Indeed, even if such works did not directly analyze the electrical conductivity of the fluids, they had an outstanding influence on EDM since they decreased plasma resistance, favored discharge occurrence, and impacted the MRR and tool wear. Moreover, additional chemical reactions and phenomena, such as electrolysis, must be examined when using water-based fluid to evaluate EDM efficiency. In this regard, it is underlined that MRR and TWR improvement and geometrical accuracy can be accomplished on the condition that process energy must be set as low as possible. 

Dealing with the micro-EDM process, though, introduces more complexity to using electro-conductive fluid, as the specific workpiece and tool material compositions, smaller dimensions, smaller sparking gaps, higher energy density, and nonnegligible effects of ions entail more significant variability and less predictability of the performance compared to its macro counterpart [12]. 

Maccarini et al. [13] proposed a study of the influence of different workpieces (AISI316L and Ti-6Al-4V), tool materials (brass and WC), and dielectric fluids (demineralized water, soya oil, and kerosene) on micro-EDM drilling performance. The ANOVA analysis comprised the variation of the current (70 and 90 as an index) and open voltage (100 V and 110 V) for evaluating the MRR, TWR, diameter overcut, and taper rate; instead, frequency (150 kHz), pulse-on time (3 µs) and gap (30 index) were kept unvaried. One of their main results declared that demineralized water significantly improved the machining speed and the MRR compared to the other dielectrics. At the same time, the TWR was consistently reduced to the detriment of micro-hole geometrical accuracy. Furthermore, the findings obtained with the regression model developed by the authors led to the conclusion that the workpiece and electrode influence on performance can be imputed to the material erosion resistance. In contrast, the effect of the dielectrics was not uniquely determined and required further investigation.

In [14], Ni et al. investigated crater size dependency on open voltage (between 30 and 90 V) and capacitance (between 0.1 and 1000 nF) in single-discharge micro-EDM using deionized water. The main results evidenced how open voltage, capacitance, and discharge energy affected the crater’s width and depth. Since a single discharge approach was considered, the impact of electro-conductivity was not directly discussed to provide evidence of the actual role of such fluid in crater formation. 

Micro-EDM performance with deionized water was also included in the analysis by Tiwary et al. [15]. In this work, the micro-EDM-induced drilling of holes in the Ti-6Al-4V workpiece was performed using DEF-92 hydrocarbon oil, deionized water, and deionized water with Cu powder. The experimentation was carried out considering a low-energy regime, i.e., an open voltage of 50 V, a current between 0.5 and 2 A, and a pulse-on time of 8 µs. The MRR, TWR, overcut, and taper evaluation showed that Cu powder-deionized water provided better results than DEF-92 and pure deionized water. Adding Cu powder to the deionized water further enhanced the fluid’s original electrical conductivity, and this increase aided in improving the machining efficiency due to more efficient heat transfer from the tool to the workpiece during such a long pulse-on time. 

The Micro-EDM-induced drilling of holes in a Ti-6Al-4V workpiece involving tap water and various additives was also proposed by Rajamanickam et al. [16], who reported results obtained via an ANOVA analysis. The authors also considered two electrode materials for the tool: brass and copper. The chosen machining regime was set to provide low-energy pulses (an open voltage between 30 and 50 V, a current between 0.5 and 2 A, and the pulse-on time was set to 100–300 µs, with a duty cycle between 55 and 75%). In such conditions, when using the copper electrode and tap water without additional particles, the MRR and micro-hole accuracy were improved. Nonetheless, the authors pointed out that the low-energy regime was essential to achieve satisfactory performance, avoid the electrolysis effect, and prevent the appearance of secondary discharges, which could be otherwise caused by debris clustering if higher energy had been chosen.

Models and experiments concerning the micro-EDM process exploiting saline water were presented in [17]. In this work, the authors studied the effect of variable electrical conductivity (4 μS/cm, 362 μS/cm, 1106 μS/cm, and 4116 μS/cm) on the electrical breakdown of the dielectric and craters. In particular, the modeling approach provided the electron density, plasma temperature, heat flux to the anode, plasma resistance, and discharge energy by varying electro-conductivity functions. Their main results showed that an increase in the electrical conductivity in saline water caused a reduction in the minimum plasma breakdown voltage at a specified gap since the higher density of the ions lowered the plasma resistance and facilitated the discharge ignition at the beginning of the process. In other words, for a specific voltage value, a higher gap value guarantees successful sparking and, at the same time, copes with the drawback of debris particle clustering. However, increasing the sparking gap implies a reduction in the energy density and a decrease in the spark efficiency. Hence, reduced crater sizes were expected. In this regard, a model proposed in [18] explained that the plasma channel forming in deionized water has a stronger pressure and radially expands outward because of vapor bubbles. As a result, the plasma channel is less confined, and the electric field distribution is widened, thus affecting the crater dimension. 

By inspecting the tendency of all the findings about micro-EDM with deionized or saline water, it is straightforward to conclude that micro-EDM performance is subject to water-based fluid characteristics, such as electrical conductivity, dielectric strength, and chemical composition. In this scenario, chosen electrode materials, process energy, and pulse duration are also paramount, and the results hint that low-energy processes are preferred. Our previous work [19] illustrated preliminary results about the micro-EDM process and performance with water-based fluids, including Garnet particles, with variable electro-conductivity. The investigation was performed in roughing and semi-finishing regimes involving high voltage and current values with variable pulse-on times and frequencies. The experimental results showed that as the electrical conductivity decreased, the MRR generally increased, whereas the TWR decreased dramatically. 

It is worth stressing that the constant polarization of the electrodes, temperature, and discharge conditions can trigger electrolysis, inducing metal corrosion and dissolution in micro-EDM with water. To cope with such issues, some authors have proposed valuable solutions, such as a new high-frequency bipolar pulse generator involving nanosecond pulses [20] and enabling the alternation of electrode polarization; this strategy proved to remove electrolysis in micro-EDM drilling using deionized water. Following a similar approach, a short-voltage pulse generator capable of reducing electrolysis in deionized water was developed in [21], where the authors proposed a model of the electrochemical reaction, conceived as a series of parallel plate capacitors and a resistance. Their results showed that if the pulse width of the micro-EDM process is significantly smaller than τ (the charging time of the circuit), the corrosion of the electrodes and consequent dissolution of metals due to electrolysis can be effectively suppressed with no need for polarization inversion. Moreover, it was observed that when very short pulses are considered (less than a hundred nanoseconds), the material removal process of micro-EDM can be considered as ablation rather than melting and evaporation [12].

Diffferently from what was already acknowledged, the present work aimed to investigate the effects of deionized and tap water fluid used in the micro-EDM process by applying a semi-finishing regime involving high voltage and current values and reduced pulse-on times and duty cycles (short pulses). Numerical analyses using COMSOL Multiphysics were proposed to estimate the temperature, plasma and electric field distributions, and crater sizes by varying the input current values, gap sizes, electrical conductivity, and permittivity of the fluids. It is worth underlining that the role of permittivity was systematically neglected in the literature; however, this characteristic impacts plasma formation, electric field distribution, and consequent crater size estimates. The experimental results involving drilled micro-holes and milled slots on the AISI 301 thin plate were finalized to evaluate the MRR, TWR, radius overcut, and surface roughness as a function of voltage and electrical conductivity (water type). Moreover, the voltage and current waveforms monitored during the trials were analyzed to discuss the micro-EDM results, especially concerning the TWR. Finally, the crater dimensions and shapes were characterized via a confocal microscope and compared to the estimates obtained from the simulations.

## 2. Machine and Experiment Set Up

**Figure 1:**
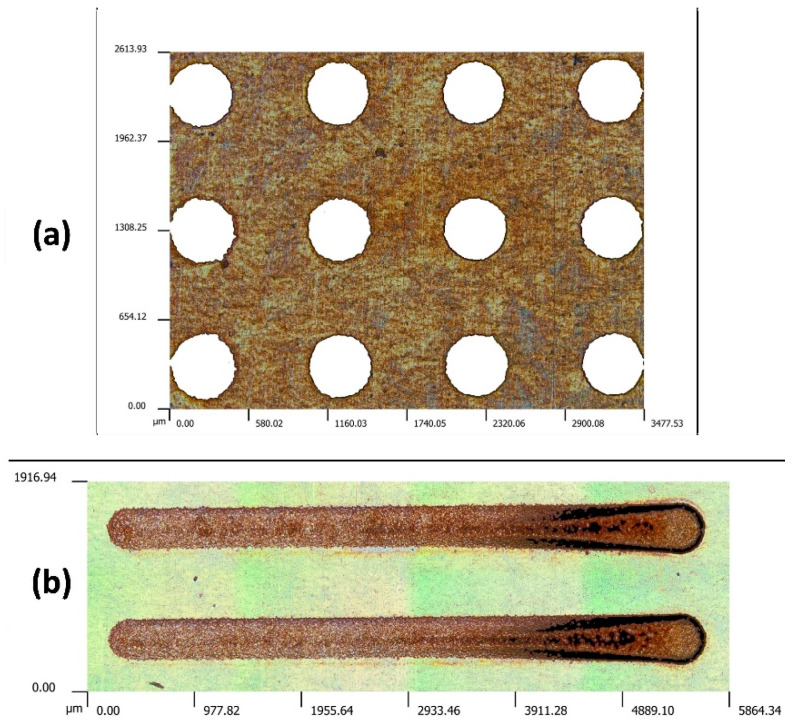
(**a**) Micro-holes machined via micro-EDM drilling and (**b**) channels machined via micro-EDM milling.

The micro-EDM machine used was the SARIX SX 200 HP (SARIX SA, Via Serrai 12, 6592 Sant’Antonino, Switzerland). Semi-finishing (E206) using an RC-type generator was set to drill holes and mill micro-channels in hardened steel (AISI 301) plates with a thickness of 0.25 mm (Figure 1). The tool electrode was a WC cylindrical rod with a diameter of 0.4 mm. The tool was negatively polarized (anode), and the workpiece was positive (cathode). As widely acknowledged, the tool’s straight polarity (negative polarity) is standard when the discharge duration is below a micro-second. Moreover, this setting is the most reasonable considering the theory of dominant electron bombardment in micro-EDM. Deionized and tap water were considered as the flushing fluids in the experiments.

Table 1 lists the micro-EDM process parameters used for the experiments: open voltage, current (maximum peak), pulse-on time, frequency, and gap (an index indicating the target distance the machine keeps to ensure stability during the process). The set pulse-on time (Ton) value is the lowest permitted by the micro-EDM machine; the frequency increases as high as possible to achieve duty cycle minimization (25%). According to the reported literature [20,21], this choice was intended to minimize water electrolysis. The experimental plan envisaged three trials for each parameter combination, which involved an open-voltage variation from 100 to 130 V.

**Figure 2:**
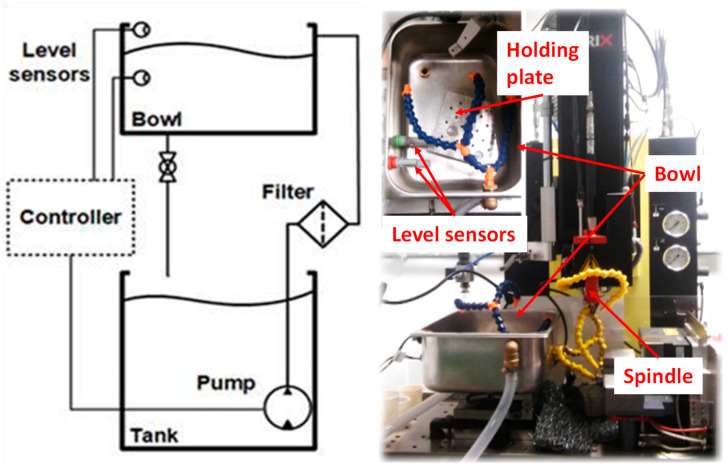
Schematic diagram of the closed-loop hydraulic auxiliary plant designed for the SARIX SX 200 HP for water-based fluids and a picture of the system installed onboard.

To separate the water-based liquids from the closed loop engaged for the flushing of hydrocarbon oil and, thus, to avoid their mutual contamination, an additional hydraulic circuit was explicitly conceived to enable fast-source liquid exchange, as evidenced in the scheme reported in Figure 2. In such an auxiliary plant, an aluminum bowl is installed on the top of the machine plate to fix the machining piece. An additional holding plate is placed within the bowl and equipped with a high-precision mechanical connection with the machine’s original plate; this connection can ensure parallelism and enables the correct reference of the workpiece. An external tank hosts the liquid used in the process, with high accessibility to its flow and charge. A submersible pump supplies the liquid into the bowl, where two level sensors control the liquid level; the pump is controlled accordingly with on/off logic through a programmable Arduino-based controller. 

Water fluids’ electrical conductivity (σ) at room temperature was measured using a conductivity probe (Primo Conductivity and TDS Testers, Hanna Instruments™, Hanna Instruments s.r.l., Viale delle Industrie, 11-35010, Villafranca, Padovana (PD), Italy) and is reported in Table 2. The measurements were performed before and after each test, and no significant variation in the conductivity was appreciated in both cases. 

### Micro-EDM Simulation: Plasma Channel Distribution and Removed Material Area Prediction 

The numerical modeling of the micro-EDM process was performed to predict the plasma channel, the electric field, the temperature distributions, and the area of the melted material. To this aim, the 2D frequency domain model was implemented in the plasma module of COMSOL Multiphysics. Following the path reported by the previous literature [22,23,24,25,26], the study was carried out by coupling the electromagnetic and heat transfer apparatus to the fluid modules. Differently from the literature, the permittivity of deionized and tap water was also included in the model. The tool and the workpiece materials were modeled considering the electromagnetic and heat data of tungsten carbide (WC) and AISI 300 steel, respectively. To evaluate the plasma channel formation, the following assumptions were applied to simplify the model:

- For the channel plasma formation, the local thermal equilibrium (LTE) condition was set;
- During the duration of the applied pulse, only a single discharge occurred in the gap;
- The dielectric fluid (water) was incompressible;
- The initial value of the electrical conductivity and relative permittivity of the water were adequately set as per the experiments (σ_Deio_ = 50 µS/cm and ε_r_deio_ = 26 for deionized, σ_Tap_ = 600 µS/cm and ε_r_tap_ = 78 for tap water);
- Flushing was assumed to remove all the debris;
- Bubble formation, ion migration, and their influence on the process were neglected.

Subsequently, stationary frequency parametric analyses were implemented by setting a frequency of 150 kHz and varying the current values (5 A, 7 A, and 10 A), the electrical conductivity and permittivity of the fluids, and the gap (e.g., the distance between the tool tip and the workpiece surface from 0.03 mm to 0.06 mm).

**Figure 3:**
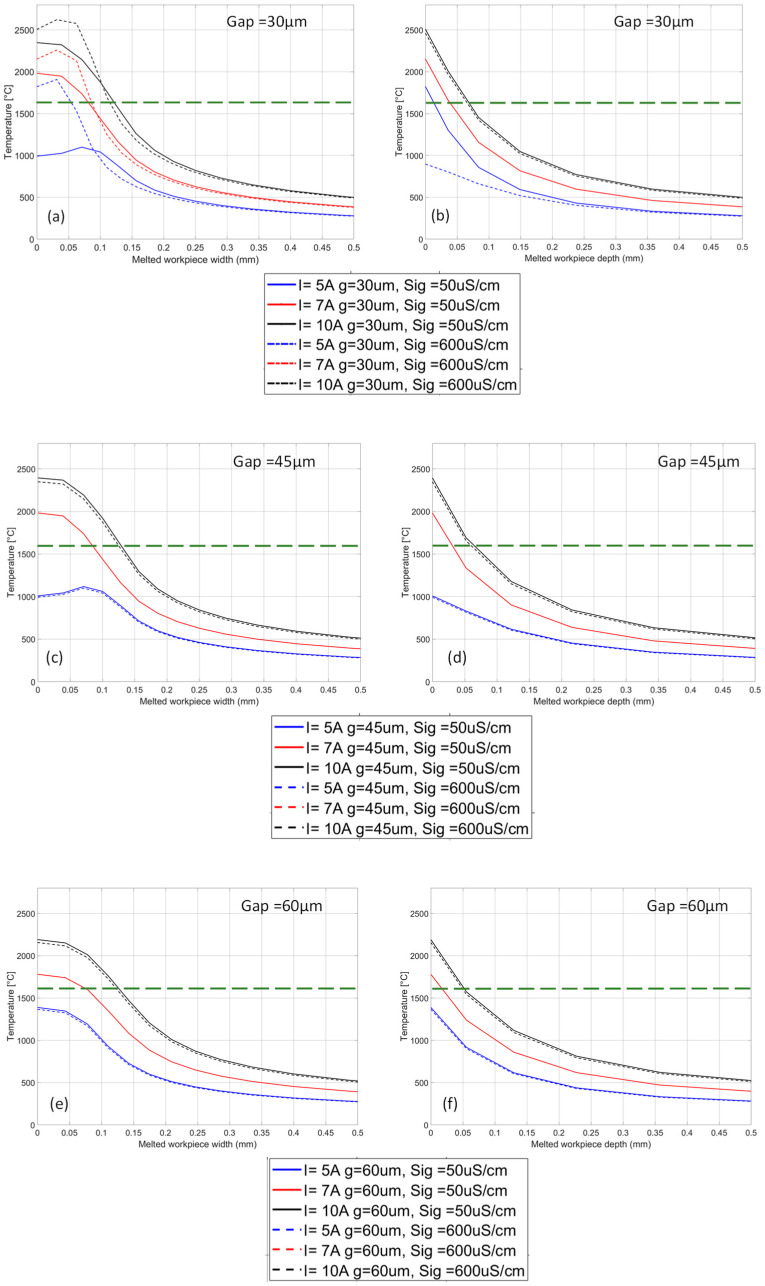
Temperature vs. width for F = 150 kHz, I = 5, 7, 10 A and variable σ for (**a**) gap = 30 µm, (**c**) gap = 45 µm, and (**e**) gap = 60 µm. Temperature vs. depth for F = 150 kHz, I = 5, 7, 10 A and variable σ for (**b**) gap = 30 µm, (**d**) gap = 45 µm, and (**f**) gap = 60 µm. The dashed green line in all plots indicates the T = 1530 °C value, corresponding to the AISI steel’s initial melting temperature.

Figure 3 depicts the temperature profiles calculated on and into the workpiece surface, considering different currents and gap width values. The different colors refer to the current amplitude, while the line type indicates the electrical conductivity (water type). The dashed green line indicates the T = 1530 °C value, corresponding to the AISI steel’s initial melting temperature. Figure 3a,b shows that when deionized water is used for a gap that is 30 µm, the workpiece melts at every applied current; for tap water, no melting is observed for current values lower than 7 A. This trend is exacerbated when the gap increases (Figure 3c–f) since the overall temperature reasonably decreases; indeed, as inferred from the remaining plots, in these conditions, no melting is observed for I < 7 A for both water fluids. It is worth stressing that the temperatures calculated for I = 7 A have close values and trends for both water fluids (the solid red and dashed lines). Therefore, the electric field distribution was investigated to compare the differences between the two cases.

**Figure 4:**
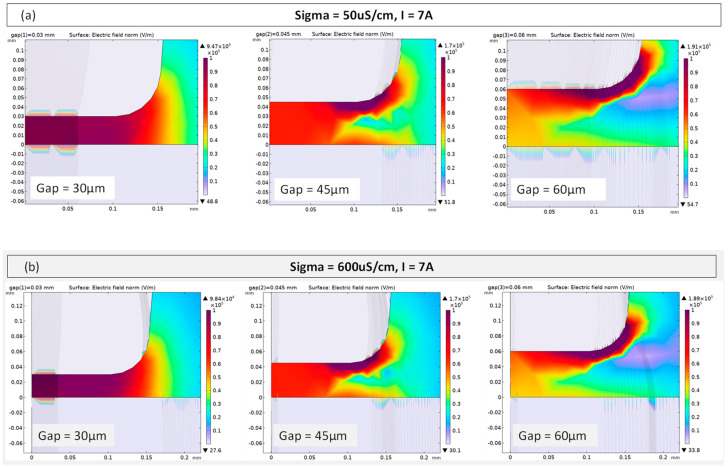
Electric field norm distribution for F = 150 kHz, I = 7 A, and for different (**a**) σ_Deio =_ 50 µS/m (deionized water) and (**b**) σ_Tap_ = 600 µS/m (tap water) as the gap is varied.

Figure 4 depicts the E-field distribution and intensity calculated for I = 7 A for all water fluids. Especially for a gap that is 30 µm, it can be noticed that, for 600 µS/cm, the E-field strength indicated by the streamlines is higher within the gap compared to 50 µS/cm, and this is also due to the higher relative permittivity associated to the higher electrical conductivity. When the gap increases, the E-field distribution changes and becomes more intense closer to the tooltip’s edge, decreasing its strength within the gap. Nonetheless, higher electric field intensity was recorded throughout the gap area when tap water was considered. These observations suggest that the crater volume would follow an inverse proportionality with the electrical conductivity since the permittivity also plays a role in plasma channel confinement.

It is worth stressing that the plasma channel distribution and the recorded temperature in the numerical domain do not exhibit significant modifications by varying the electrical conductivity or further increasing the current’s values. This asymptotic behavior could be ascribed to the model limitation of some temperature-dependent parameters, such as temperature-dependent electrical conductivity.

**Figure 5:**
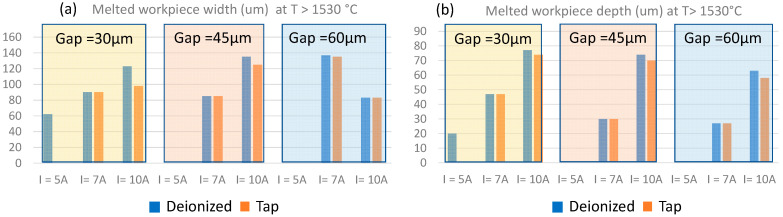
The melted workpiece material area, (**a**) width, and (**b**) depth calculated at T > 1530°C.

The width and depth of the melted material are reported in Figure 5 and are calculated for all gap and current values considering the area identified by the isothermal curves for T > 1530 °C. As inferred from the histograms, the width of the melted area is more influenced by the current’s values, as expected. Still, it exhibits a variability ascribed to electrical conductivity and permittivity: indeed, for higher current values, the melted area generally increases in width and depth considering deionized water, whereas the width of the melted area is lower for tap water, indicating that in this latter condition, a smaller crater size is obtained due to higher permittivity, which improves electric field confinement. 

For clarity, the calculated width and depth of the melted material do not correspond to the actual crater’s size. The numerical results provide reasonable qualitative indications for the expected experimental outcomes. A more accurate estimate of crater size could be obtained by using thermo-fluidic analysis that considers plasma pressure, including material evaporation, resolidification, and the melt-pool effect in the model [26]. Furthermore, the model does not consider that part of the energy is exploited for electrolysis in the micro-EDM process. Finally, a less-than-a-microsecond pulse-on time and the high frequency set for the trials require time-dependent analysis to inspect appropriately the plasma breakdown.

## 3. Micro-EDM Experiments: Results and Discussion

### 3.1. Micro-EDM Performance: Evaluation of the MRR, TWR, and Sparking Gap in the Applied Open Voltage and Dielectric Fluid Type Function

**Figure 6:**
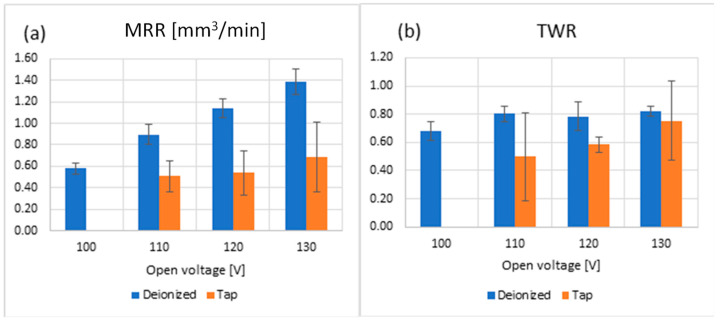
(**a**) MRR, (**b**) TWR, vs. open voltage performed in the semi-finishing regime for each fluid type.

The average values and standard deviation of the MRR and TWR are reported in Figure 6 as a function of the type of water fluid. Figure 6a shows that, for deionized water, the MRR increases linearly and significantly with the applied open voltage. When considering tap water, the micro-EDM process did not start at Vo = 100 V, corresponding to a current peak of 5 A. This result agrees with the simulation results involving an I = 5 A current, where no built plasma and material melting was observed. Furthermore, considering higher open voltages, the MRR shows a minor increase for tap water compared to deionized water. The explanation for such behavior can be found in the chemical reactions occurring for the tap water trials, especially witnessed by the oxidation layers deposited on the tool. Figure 6b reports the TWR, average, and standard deviation versus the open voltage and type of dielectric fluids. The TWR is the ratio between the volume of material removed from the tool’s electrode and the volume removed from the workpiece. It can be observed that deionized water exhibits a TWR greater than tap water, with a slight variation as the open voltage (and current) increases. The difference can be ascribed to the chemical composition of the fluids.

**Figure 7:**
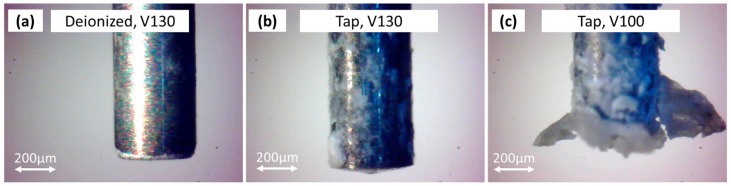
Electrode tool after erosion with (**a**) deionized water and Vo = 130 V, (**b**) tap water and Vo = 130 V, (**c**) and tap water Vo = 100 V.

Comparing the three images reported in Figure 7, it is possible to notice that the tool’s electrode presents a deposited oxidation layer, mainly affecting the lateral tool surface when tap water is used. The thickness of the deposited layer increased with the erosion time, thus leading to a change in the tool’s electrical conductivity and, consequently, reducing the tool’s wear. This fact also involved a reduction in workpiece material removal. Additionally, when using tap water and applying an open voltage of Vo = 100 V, the deposition layer on the tool surface became relevant, so much so that it could prevent a spark from occurring. It can also be noted that part of the energy devoted to the process was wasted with the ignition of the chemical reactions and consequent electrolysis. Therefore, the machining efficiency in terms of the MRR was decreased. 

**Figure 8:**
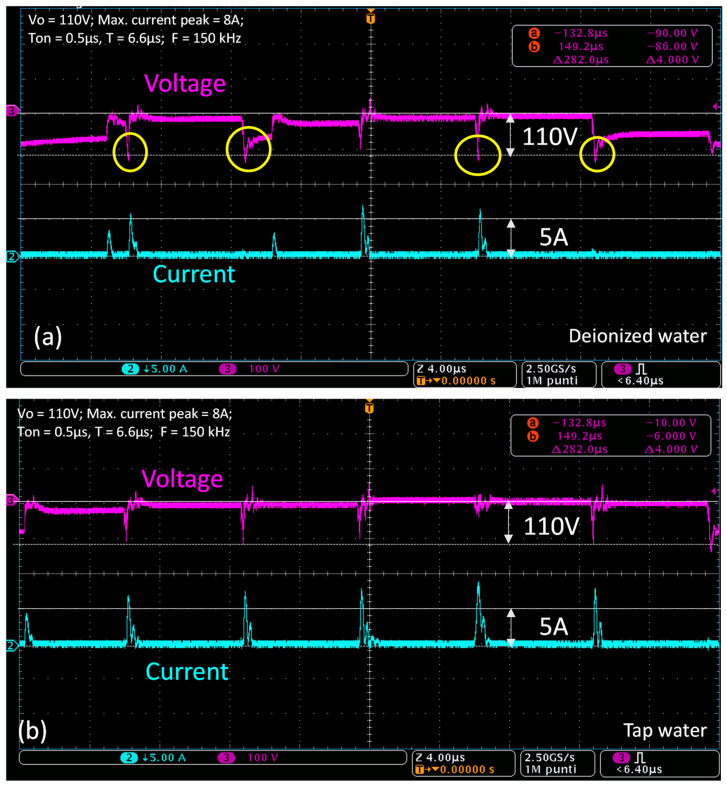
Voltage and current waveforms monitored on the MDO3001C machine during hole drilling with (**a**) deionized water and (**b**) tap water, considering Vo = 110 V, maximum current peak = 8 A, pulse-on time = 0.5 µs, and a period T = 6.6 µs (F = 150 kHz). The yellow circles underline that, when using deionized water, the voltage waveform goes below zero in the breakdown phase. The waveforms were visualized with a high resolution obtained with 2.5 GS/s and a magnified window of 40 µs. The time trigger was set to <6.4 µs, the current was 5 A/div, and the voltage was 100 V/div.

The voltage and current waveforms were also monitored during the trials to inspect micro-EDM evolution (Figure 8). The monitoring setup was composed of the Tektronix MDO4000C oscilloscope (Tektronix Srl, Via del Lauro 9, 20121 Milano MI, Italy), one passive voltage probe TPP0201 Tektronix (Tektronix Srl, Via del Lauro 9, 20121 Milano MI, Italy), and a TCP312A Tektronix (Tektronix Srl, Via del Lauro 9, 20121 Milano MI, Italy) current probe connected to a Tektronix TCPA300 amplifier (Tektronix Srl, Via del Lauro 9, 20121 Milano MI, Italy). The waveforms were visualized considering a high resolution obtained with 2.5 GS/s and a magnification window of 40 µs. The time trigger was set at <6.4 µs, while the current values were depicted with 5 A/div and a voltage of 100 V/div. As visible in Figure 8a, in the case of deionized water, when discharge happens, the voltage drops (breakdown phase) and goes slightly below zero before it rises and settles again to Vo. Therefore, during the breakdown phase, the V waveform changes its sign. Additionally, the current oscillations were found to be slightly negative in this case, as evidenced by the yellow circles in Figure 8a. Hence, the tool’s and electrodes’ polarities were assumed to be quickly inverted. This dynamic has a two-fold consequence: the electrolysis is slightly minimized at the expense of tool wear increase induced by alternate current behavior [27]. Conversely, considering tap water, this behavior is not observed in Figure 8b, where the V waveform drops during the breakdown phase, barely approaching 0 V. In this case, the current waveform does not exhibit appreciable oscillations below zero, so no clear evidence of alternate current behavior can be assessed. This, in turn, can explain most of the oxide layer deposits on the tool and tool wear reduction. This means that erosion in tap water always occurs in constant electrode polarization, thus leading to electrolysis more than in the case of deionized water. 

**Figure 9:**
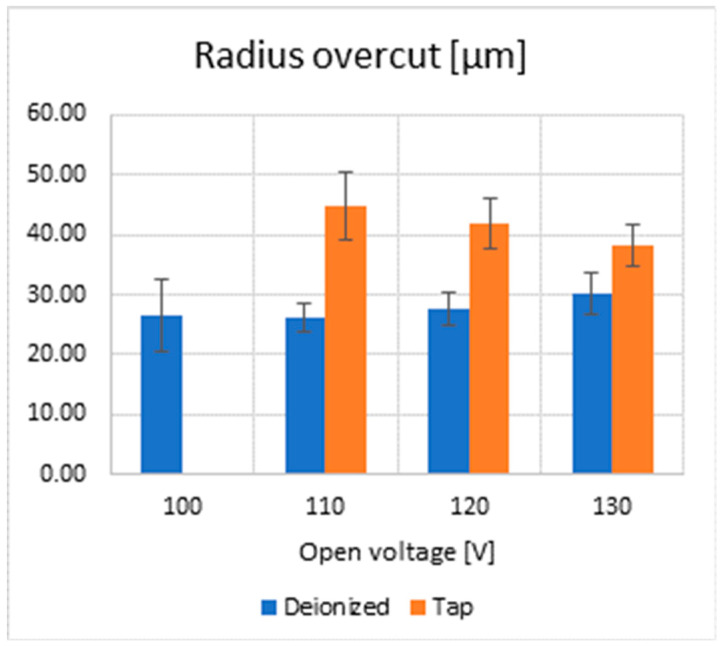
Radius overcut vs. open voltage performed in the E206-semi-finishing regime for each fluid type.

The average values and standard deviations of the measured radius overcuts are depicted in Figure 9. The radius overcut is the difference between the eroded hole and the tool’s electrode radius measured during drilling. As inferred from the diagram, the value settles around 28–30 µm for deionized water and slightly increases as V increases. Conversely, when the tap water fluid is used, it decreases from 45 to 38 µm as V increases. Generally, when the fluid’s electrical conductivity grows, the gap and radius overcut are expected to increase. Indeed, the ions diffused in the water, those added by the material removal process, and the absorption of carbon dioxide (CO_2_) contributes to further enlarging the gap. The radius overcut for tap water indicates the opposite tendency, though. This variability can be reasonably ascribed to the reduced energy of the process due to electrolysis and the oxidation layer deposited onto the tool surface, which hinders the discharge and increases the erosion time.

### 3.2. Crater Size and Surface Roughness Evaluation

**Figure 10:**
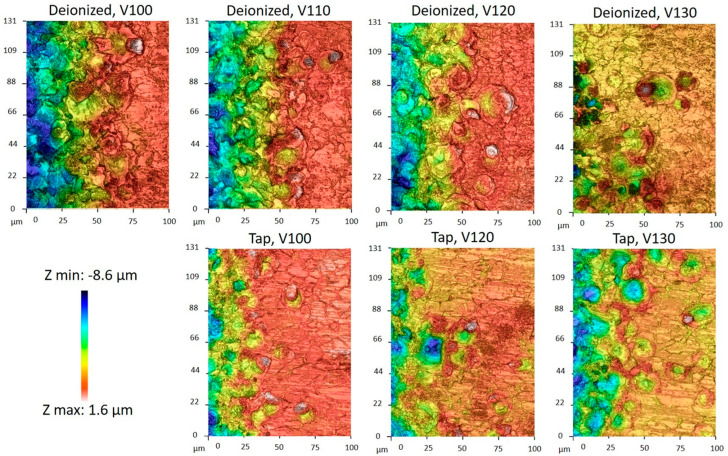
Confocal microscope acquisitions: crater shape considering different dielectrics and machining regimes.

The crater diameters and the surface roughness Ra were estimated via optical Profiler Sensofar S Neox 3D (SENSOFAR, Parc Audiovisual de Catalunya Ctra. BV-1274, KM 108225 Terrassa (SPAIN)) used in the confocal mode. Figure 10 depicts the acquisitions of the border machine surface, where single craters are more evident, showing the crater morphology for the different fluids. 

In order to preliminarily evaluate the relationships between the crater sizes, open voltage, and fluid types, 25 craters were manually identified for each set of parameters and were measured. The average and standard deviation diameters of the crater size measurements are listed in Table 3. Even though the standard deviation is quite large, some trends can be observed: indeed, the craters become more significant as voltage and current increase. Furthermore, as anticipated with the simulation results, the mean values of the crater sizes are larger for deionized water than for tap water.

**Figure 11:**
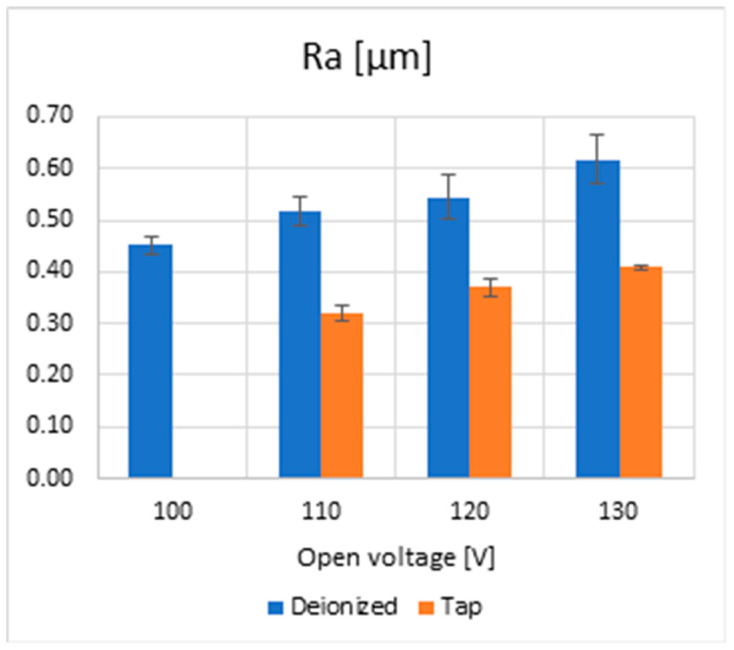
Surface roughness, Ra, for the semi-finishing regime vs. the open voltage type of fluids.

From Figure 11, it can be noticed that surface roughness shows a similar trend for both fluids and increases with open voltage. Although there is not such a big difference in crater size considering deionized or tap water, Ra is improved when using water with higher electrical conductivity. This result leads to two primary considerations: the first is that tap water allows the formation of shallower craters compared to deionized water. The second consideration is related to why this happens: indeed, as already discussed for the MRR, part of the energy devoted to the process is wasted by chemical reactions, leading to consequent electrolysis. Less energy involves a lower MRR and shallower crater depth, which implies a lower Ra. 

## 4. Conclusions

In this work, micro-holes and slots realized in hardened thin steel plates were machined via micro-EDM, exploiting fluids with different electrical conductivity: deionized and tap water. The micro-EDM trials were carried out without tool wear compensation during milling. The processes were performed by using a semi-finishing regime with short pulses (F = 150 kHz, Ton = 0.5 µs, and a duty cycle of 25%). The electrical conductivity of the water was also measured with a probe. 

COMSOL Multiphysics simulations in the frequency domain verified the process’s feasibility and the plasma formation, electric field, and temperature distributions as water-based fluid’s frequency, electroconductivity, permittivity, applied current, and tool-workpiece distance (gap) varied. The main results evidenced that higher electrical conductivity and permittivity hindered plasma channel formation and prevented material melting for low current values. Moreover, the predicted melted workpiece area was higher for deionized water than tap water. The proposed numerical results provide reasonable qualitative indications for the experimental outcomes. However, the estimated width and depth of the melted material cannot correspond to the actual crater size due to model assumptions and neglected ions and electrolysis phenomena. 

Experimentally, the evaluation of the MRR trends as a function of the electrical conductivity of the fluids underlines that a higher electrical conductivity fluid, such as tap water, induces a lower MRR. Additionally, in this case, when voltage and current are increased, the MRR exhibits a minor increase compared to the deionized case. On the contrary, the TWR is further minimized by considering tap water, likely due to the oxidation layer deposited on the tool surface and induced by electrolysis. The erosion process for tap water only occurred for sufficiently high open voltage and current values, particularly Vo < 110 V and I < 7 A for the proposed parameter setting. This result also aligned with the numerical ones involving lower energy process modeling. In summary, the MRR and TWR generally displayed lower values for tap water than deionized water because of electrolysis, which uses part of the process energy. 

Regarding the radius overcut analysis, it was found in the trials with tap water that this factor was broader than that evaluated for deionized water and decreased as open voltage increased. This result is in contrast to what is currently acknowledged by the literature for EDM processes exploiting tap water as a dielectric since higher gap values should be generally expected. The evaluation of the crater led to the preference for deionized water to attain larger sizes. This result agrees well with the simulation outcomes. Nonetheless, it was also observed that surface roughness, Ra, could be reduced using higher electrical conductivity fluids such as tap water rather than deionized water; the motivation of such behavior is ascribed to chemical reactions causing electrolysis, which decreases the energy involved for the erosion and yields shallower craters and thus a better Ra. 

## Figures and Tables

**Table 1:** Micro-EDM process parameters.

	Open Voltage, Vo (V)	Current (Index)	Pulse-On Time (µs)	Frequency (kHz)	Gap (Index)
E206 (semi-finishing)	100, 110, 120, 130	50 (peak: 5, 7, 8, 10 A)	0.5	150	65

**Table 2:** Electrical conductivity, “σ“ measurements, of the water-based fluids at room temperature.

	Deionized Water	Tap Water
Electrical conductivity—σ (µS/cm)	50	600 (*)

(*) Chemical composition (mg/L): Ca++ 96.8; Mg++ 43.6; Na+ 19.9; K+ 2.48; HCO_3_ 477; Cl 32.3; F 0.18; SiO_2_ 16.2.

**Table 3:** Crater diameters (mean and standard deviation values) for deionized and tap water for different process parameters.

Open Voltage (V)/Current (A)	Average Diameter (µm)	Standard Deviation Diam (µm)	Average Diameter (µm)	Standard Deviation Diameter (µm)
	Deionized Water		Tap Water	
100/5	15	±14	--	--
110/7	18	±4	17	±3
120/8	20	±6	19	±4
130/10	21	±7	20	±7

## Data Availability

Data are contained within the article.
